# The Antiseptic Treatment of Typhoid Fever

**Published:** 1898-05-21

**Authors:** 


### THE ANTISEPTIC TREATMENT OF TYPHOID FEVER.

A long article by Brig.-Surg.-Lieut.-Colonel Quill in the *British Medical Journal* is devoted to the treatment of enteric fever in India on what is called "the antiseptic principle." Putting on one side the diet, which we should say consisted of milk given in somewhat smaller quantity than is usual here, along with a pint of beef tea or chicken broth per diem, the antiseptic method employed would seem to have consisted of "the production and maintenance of intestinal antisepsis." The antiseptic usually employed was Calvert's No. 1 carbolic acid given in three minim doses every second or third hour—that is, five or six doses in mild cases, and ten doses in severe cases in the twenty-four hours. The carbolic acid was given in combination with tinct. chloroformi co., tinct. cardamomic co., syrup aurantii, and aqua chloroformi, a mixture which is maintained to be "not only efficient in an antiseptic sense, but most palatable." The cases treated numbered forty-six, and there were two deaths. This is good so far as it goes, but on the one hand it must not be forgotten how greatly the virulence of typhoid fever varies at different times, and on the other we must not omit to notice that the cases were evidently treated with care and judgment in other respects, such as the use of cold baths and ice-packing when required, besides the giving of the antiseptics, so that we do not feel drawn to argue very strongly in favour of the "antiseptic" part of the treatment from the small number of cases given.

